# Layer-by-layer siRNA/poly(L-lysine) Multilayers on Polydopamine-coated Surface for Efficient Cell Adhesion and Gene Silencing

**DOI:** 10.1038/s41598-018-25655-7

**Published:** 2018-05-17

**Authors:** Cheol Am Hong, Ho Yeon Son, Yoon Sung Nam

**Affiliations:** 1 Department of Materials Science and Engineering, Korea Advanced Institute of Science and Technology, Daejeon, 34141 Republic of Korea; 2 KAIST Institute for the NanoCentury, Korea Advanced Institute of Science and Technology, Daejeon, 34141 Republic of Korea

## Abstract

For tissue engineering applications, small interfering RNA (siRNA) is an attractive agent for controlling cellular functions and differentiation. Although polyionic condensation of nucleic acids with polycations has been widely used for gene delivery, siRNA is not strongly associated with cationic carriers due to its low charge density and rigid molecular structures. The use of an excess amount of cationic carriers is often used for siRNA condensation, though they can induce severe cytotoxicity. Here we introduce the self-assembly of siRNA with mild polyelectrolytes into multilayers for efficient gene silencing during cell proliferation. The multilayers were prepared through the sequential layer-by-layer deposition of siRNA and poly-L-lysine (PLL) on a polydopamine-coated substrate. The cells, grown on the siRNA/PLL multilayers, exhibited a remarkable inhibition of the expression of target genes as compared to the use of scrambled siRNA. The gene silencing efficiency depends on the number of siRNA layers within a multilayer. This result indicates that siRNA/PLL multilayers can be potentially utilized for efficient surface-mediated siRNA delivery.

## Introduction

RNA interference (RNAi) has been firmly established as a powerful tool to specifically inhibit the expression of target genes, which can be triggered when exogenous small interfering RNA (siRNA) is introduced into the cytoplasm^1–5^. Due to its unique roles in regulating gene expression, siRNA has been extensively explored in clinical trials for the treatment of genetic disorder and cancers^6–8^. However, siRNA itself can hardly penetrate cellular lipid membranes because of its unfavorable physicochemical properties (e.g. hydrophilicity, anionic charges and high molecular weights)^9–11^. Therefore, a safe and efficient intracellular delivery of exogenous siRNA remains highly challenging.

Among of the current strategy for gene delivery is to form compact and stable nanoparticles via electrostatic interactions between anionic genes and cationic macromolecules (i.e. synthetic polymers, peptides and lipid vehicles)^12–14^. For examples, poly-L-lysine (PLL), a cationic peptide, has been shown to be effective in condensing plasmid DNA (pDNA) to produce compact pDNA nanoparticles^15^. Unlike pDNA condensation, siRNA is not strongly associated with PLL owing to its low surface charge density and stiffness^16–18^. To form siRNA nanoparticles, it often requires the use of the excess amounts of cationic carriers with high molecular weights; however, it can also induce severe cytotoxicity^19,20^. Besides, the strong cationic surface charge of nanoparticles can induce the non-specific adsorption of serum proteins, causing the particle aggregation that is readily removed by the immune system^21^.

Layer-by-layer (LBL) self-assembly has been recognized as a simple and versatile method to fabricate thin polyelectrolyte multilayers via the sequential deposition of oppositely charged polyelectrolytes^22–25^. The LBL technique can efficiently embed various electrolytic biomolecules, including genes, proteins and inorganic particles, in a multilayer structure^26–28^. Also, the loaded molecules can be released in a sustained manner during the hydration and dissolution of the multilayer in an aqueous solution.

Polydopamine (PDA) coating has been widely used for surface modification and functionalization, especially a super-hydrophobic surface^29–33^. Like mussel adhesion proteins (MAPs), PDA displays a large number of catechol-based moieties that can strongly bind to a wide range of organic and inorganic surfaces. PDA coating can be implemented simply by dip-coating in an alkaline solution of dopamine^29^. The surface of PDA coating can serve as an anchor for the loading of functional groups, including amine- and thiol-bearing compounds via Michael addition or Schiff-based reactions^34,35^. In addition, catechol has a redox potential of +530 mV vs. a normal hydrogen electrode at pH 7, which makes PDA attractive for electrochemical applications^36–39^. For example, the PDA layer can mediate the on-surface reduction of metal precursor ions into solid nanostructures because their redox potentials are relatively higher than that of catechol. This property also allows PDA to strongly bind to various inorganic surfaces. Therefore, the PDA-coated layer can serve as an effective platform when surface modification needs to be independent of the properties of the underlying materials.

Recently, the PDA-coated substrates showed an effective immobililization of stable pDNA complexes for surface-mediated gene delivery^40^. In this study, we employed LBL self-assembly to prepare a siRNA/PLL multilayer on the PDA-coated substrate for surface-mediated siRNA delivery. The surface of siRNA/PLL multilayer induced the effective cell adhesion, spreading and proliferation without any severe cytotoxicity. Notably, the cells grown on the siRNA/PLL multilayers exhibited the remarkable inhibition of the expression of target genes. Interestingly, the gene silencing effect is correlated with the number of a siRNA layer within a siRNA/PLL multilayer.

## Results and Discussion

### Preparation of siRNA/PLL multilayers loaded on the PDA-coated substrates

**Figure 1:**
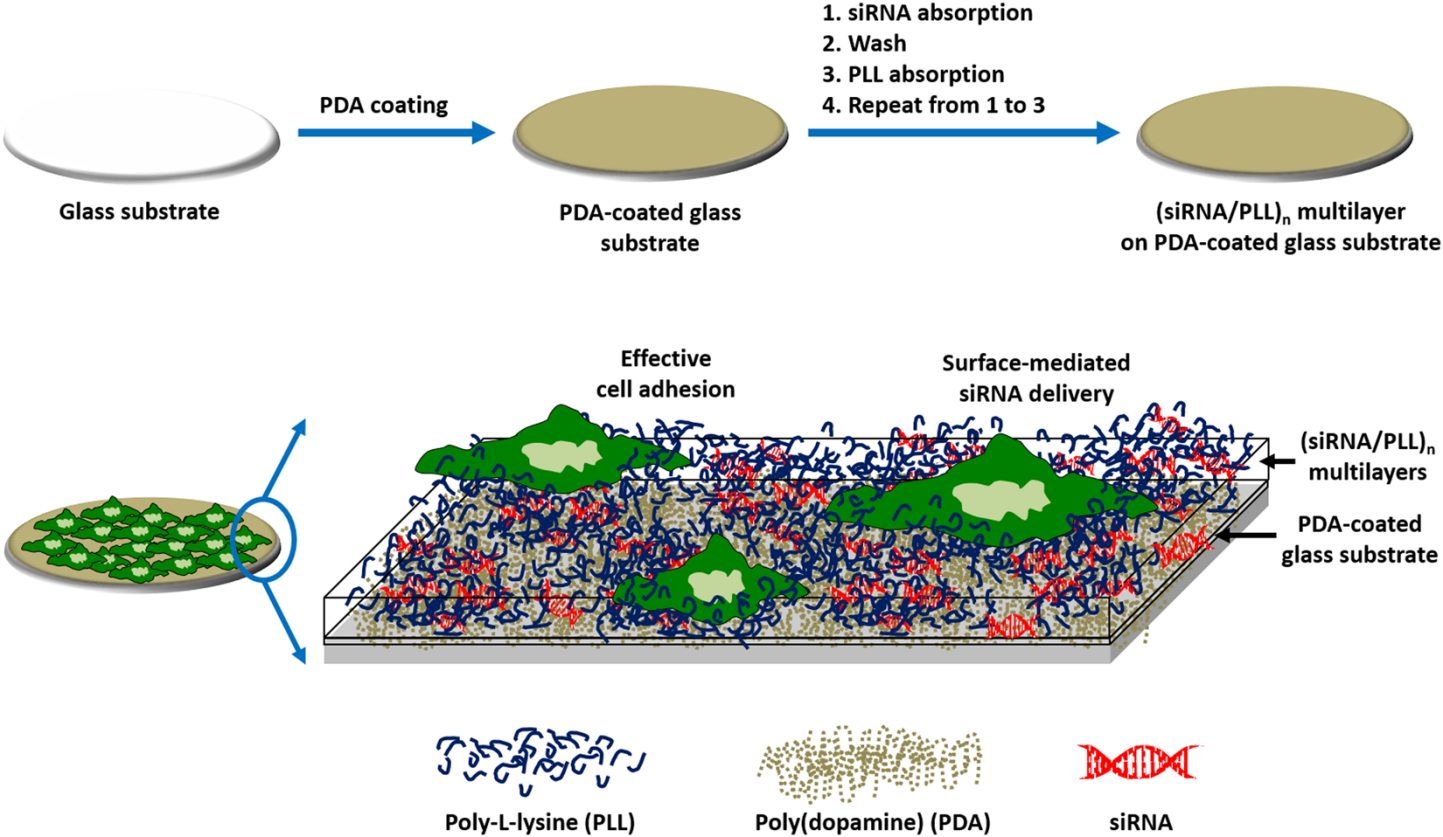
Schematic description of the siRNA/PLL multilayer on the PDA-coated glass fabricated via layer-by-layer electrostatic self-assembly, which facilitates the efficient cell adhesion and gene silencing.

The siRNA/PLL multilayer, consisting of siRNA and PLL on the PDA-coated substrate, was prepared via LBL which facilitated the effective cell adhesion and proliferation as well as gene silencing effect (Fig. 1). The number of siRNA/PLL bilayers was denoted by “n” in the (siRNA/PLL)_n_ multilayer. The surface of the glass was coated with PDA via the oxidative self-polymerization of dopamines to build up the siRNA/PLL multilayer. During the polymerization of dopamine, it is well known that there is a color change from transparent to dark on the PDA-coated surface due to the catechol oxidation^41^. The surface of glass became dark after the PDA coating process, which indicated the successful PDA coating on the glass substrate (Fig. S1). The resultant PDA-coated layer can serve as a substrate for the sequential adsorption of siRNA and PLL via electrostatic self-assembly because of adhesive functional groups on the surface of PDA coatings. Notably, the last layer of the (siRNA/PLL)_n_ multilayers was coated with PLL because PLL can facilitate the efficient cell adhesion, spreading and proliferation as well as protect siRNA from enzymatic degradation, resulting in enhanced surface-mediated gene silencing.

### Characterization of siRNA/PLL multilayers

**Figure 2:**
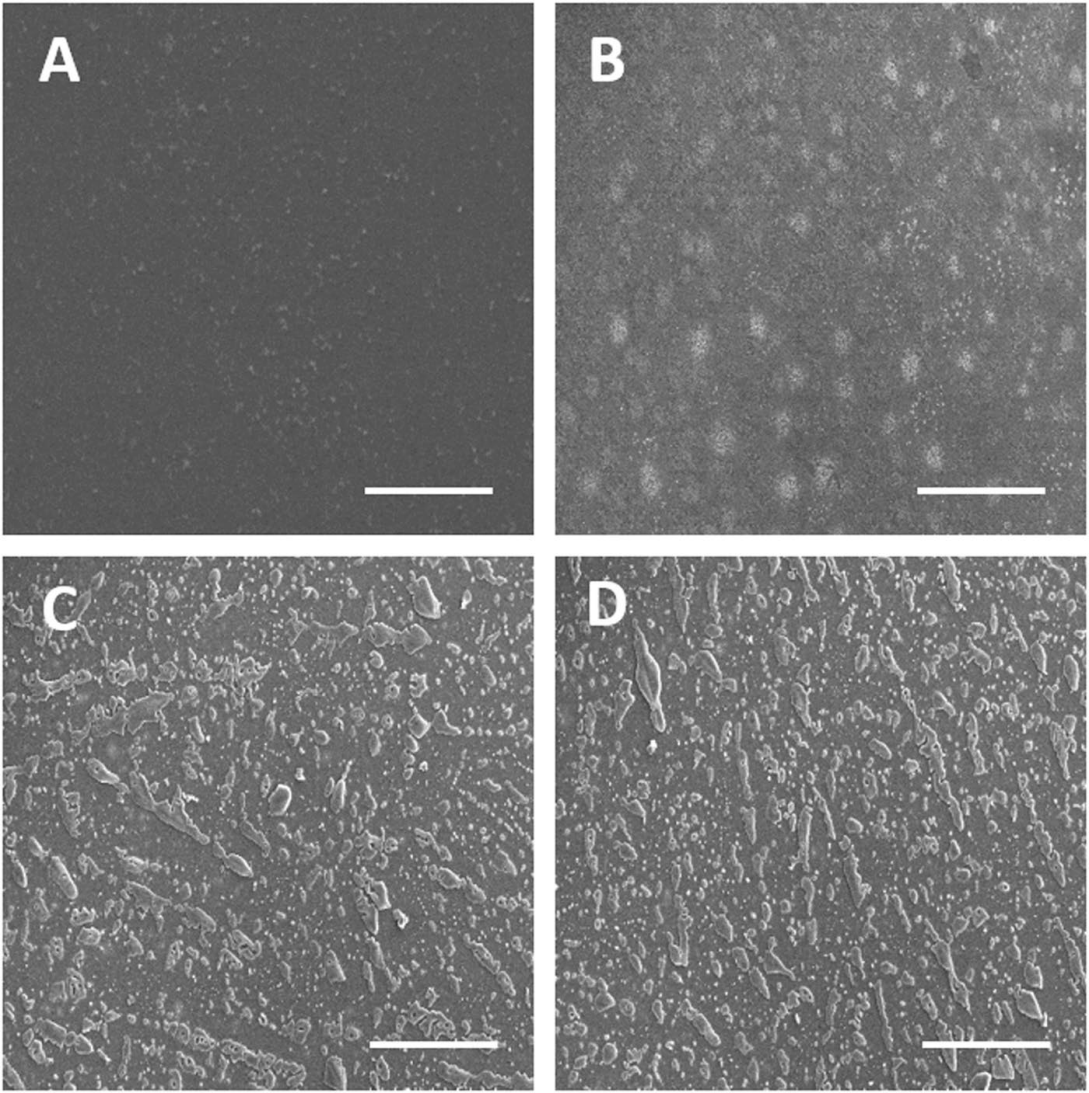
SEM images of PDA-coated glass surface (**A**), (siRNA/PLL)_1_ (**B**), (siRNA/PLL)_3_ (**C**) and (siRNA/PLL)_6_ (**D**) on the PDA-coated substrates. Scar bar = 200 μm.

The surface morphology of the (siRNA/PLL)_n_ multilayers was observed using scanning electron microscopy (SEM). As shown in Fig. 2, the surface of (siRNA/PLL)_n_ multilayer was much rougher with increasing the number of siRNA/PLL bilayers. However, the pristine glass surface did not show a roughened substrate under the same experiment condition (Fig. S2). Interestingly, the surface of (siRNA/PLL)_1_ multilayer exhibited the even distribution of aggregated particles with average sizes of approximately 900 nm, which may be attributed to the formation of siRNA/PLL complexes during LBL self-assembly process. This result suggests that siRNA molecules can be adsorbed in the form of siRNA/PLL complexes within the siRNA/PLL multilayer. Furthermore, the thickness of (siRNA/PLL)_n_ multilayers steadily increased with increasing the number of siRNA/PLL bilayers, which indicates the successful formation of (siRNA/PLL)_n_ multilayers on the PDA-coated glass surface (Fig. S3).

**Figure 3:**
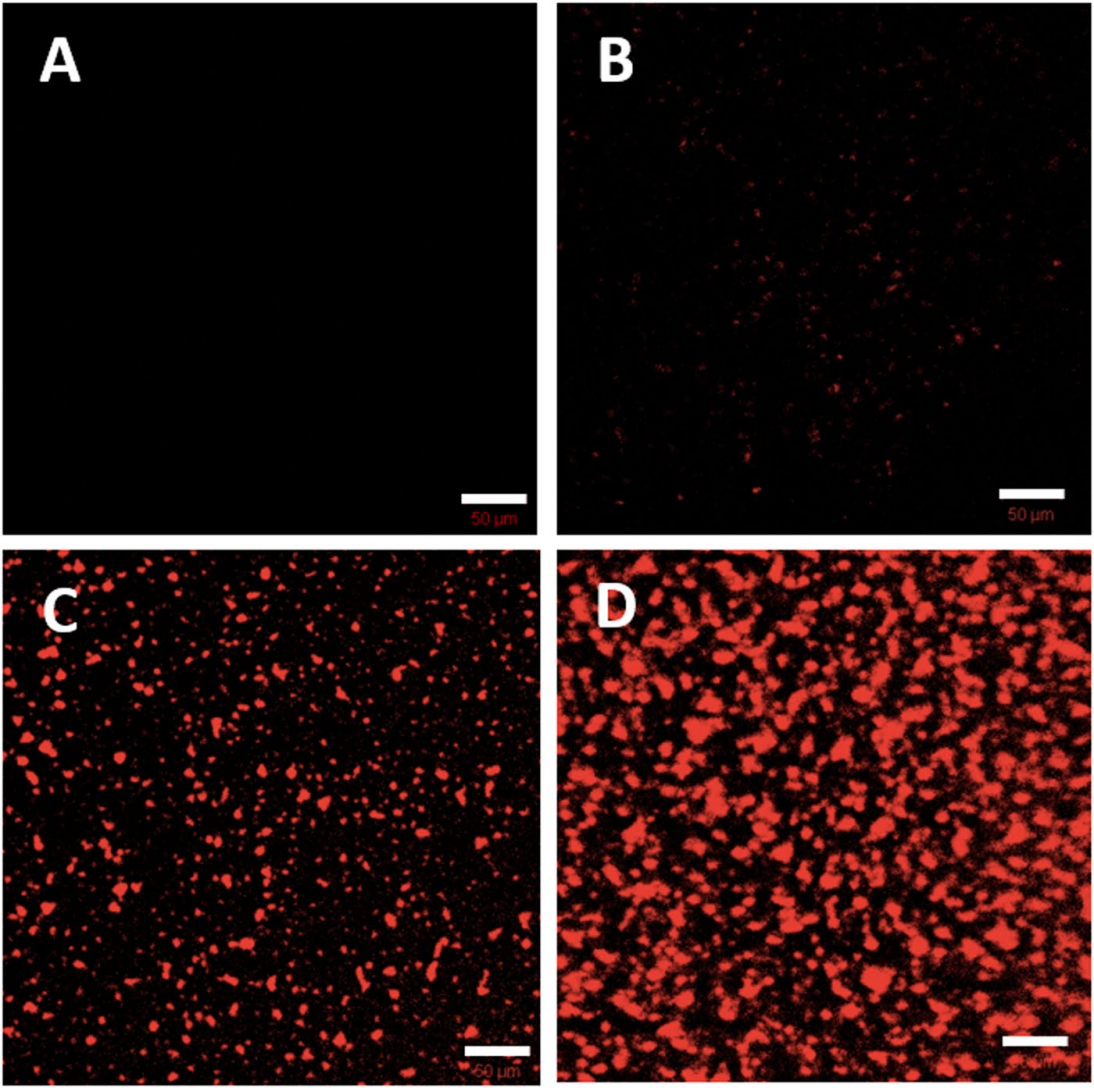
Confocal microscopy images of PDA-coated glass surface (**A**) and (siRNA/PLL)_1_ (**B**), (siRNA/PLL)_3_ (**C**), (siRNA/PLL)_6_ (**D**) on the PDA-coated substrates. Scale bar = 50 μm. siRNA is labeled with tetramethylrhodamine (TAMRA).

To further verify whether siRNA was effectively incorporated within the (siRNA/PLL)_n_ multilayer, we used fluorescently-labeled siRNA for the fabrication of (siRNA/PLL)_n_ multilayer (Fig. 3). The confocal laser scanning microscopy image of siRNA/PLL multilayers showed the scattered red fluorescent dots, suggesting the successful adsorption of siRNA molecules within the (siRNA/PLL)_n_ multilayer. Notably, the fluorescent intensity of red dots became much higher with increasing the number of a layer. The result indicates that the sequential deposition of siRNA and PLL through LBL self-assembly can be effective to form the (siRNA/PLL)_n_ multilayer on the PDA-coated surface. In addition, the loading efficiencies of siRNAs were 91.3 ± 2.8%, 93.4 ± 3.1% and 92.2 ± 2.7% for n = 1, 3 and 6 of the (siRNA/PLL)_n_ multilayers, respectively. This result indicates that the loading amounts of siRNA could be precisely controlled by adjusting the number of the siRNA layer in the (siRNA/PLL)_n_ multilayer. Notably, siRNA embedded in the (siRNA/PLL)_6_ multilayers did not release after incubated in phosphate-buffered saline (PBS) for 3 and 5 days, which can facilitate surface-mediated intracellular uptake and gene silencing (Fig. S4).

### Cytotoxicity and gene silencing efficiency of siRNA/PLL multilayers

**Figure 4:**
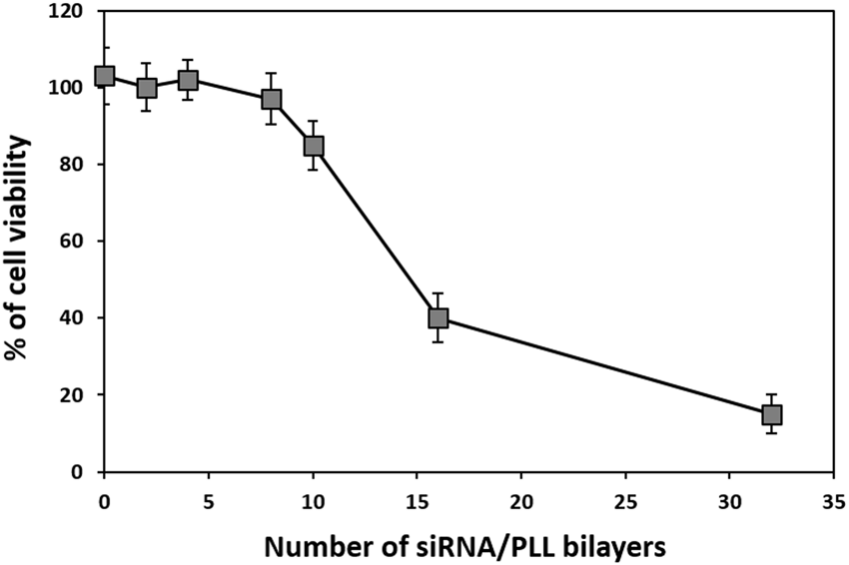
Cell viability of HeLa-GFP cells grown on the (GFP siRNA/PLL)_n_ multilayers (n = 0, 2, 4, 8, 10, 16 and 32) with increasing the number of siRNA/PLL bilayers.

The cytotoxicity of the prepared (siRNA/PLL)_n_ multilayer was determined by measuring the cellular viability of HeLa-GFP cells grown on the various (siRNA/PLL)_n_ multilayer (Fig. 4). No significant cytotoxicity was observed up to 8 layers of the siRNA/PLL bilayers, (siRNA/PLL)_8_. However, the (siRNA/PLL)_10_ multilayer decreased the cell viability to 85.2% ± 5.3%, which might be related to its higher positive surface charge. It has been known that the cytotoxicity of cationic polymers mainly depends on their high surface charge density, surface hydrophobicity and composition^42^. In addition, there was no significant difference in the adherent cell density between pristine PDA-coated glass surfaces and (siRNA/PLL)_n_ multilayers after short incubation of 3 h: 2.91 × 10^4^ ± 0.31 cells/cm^2^ (n = 0), 3.02 × 10^4^ ± 0.25 cells/cm^2^ (n = 2), 2.83 × 10^4^ ± 0.24 cells/cm^2^ (n = 4) and 2.91 × 10^4^ ± 0.15 cells/cm^2^ (n = 8) of the (siRNA/PLL)_n_ multilayers on the PDA-coated substrates (Fig. S5). This result indicates that the surface of (siRNA/PLL)_n_ multilayers did not any affect the cell adhesion as compared to that of pristine PDA-coated glass substrates. According to the results described above, we investigated the gene silencing efficiency of the (siRNA/PLL)_n_ multilayers (i.e. 1, 3 and 6) that did not show any cytotoxicity.

**Figure 5:**
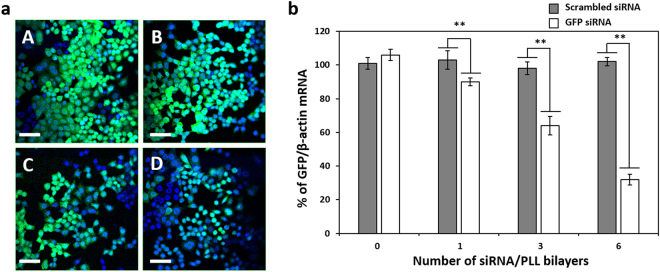
Confocal microscopy images (**a**) and target-specific gene silencing (**b**) of HeLa-GFP cells grown on the GFP siRNA/PLL multilayers. PDA-coated glass surface (A) and (siRNA/PLL)_1_ (B), (siRNA/PLL)_3_ (C) and (siRNA/PLL)_6_ (D) on the PDA-coated substrates. Scar bar = 50 μm. **P < 0.01. s.

The gene silencing of (GFP siRNA/PLL)_n_ multilayer was evaluated by the fluorescence intensity of HeLa-GFP cells loaded on various (GFP siRNA/PLL)_n_ multilayers using confocal laser scanning microscopy. Confocal images exhibited that GFP fluorescence of the cells grown on all of the (GFP siRNA/PLL)_n_ multilayers was significantly decreased compared to the PDA-coated substrate (Fig. 5a). This result suggests that siRNA/PLL multilayers provide an efficient cell proliferation and surface-mediated gene silencing at the same times. Furthermore, the remarkable reduction in the GFP fluorescence of the cells grown on all of the (GFP siRNA/PLL)_n_ multilayers exhibited no significant cytotoxicity, which indicates that the gene silencing effects were derived from the RNAi activity. Notably, the observed reduction in the GFP fluorescence depended upon the number of siRNA/PLL bilayers due to the increased amounts of siRNA adsorbed to the surface.

To confirm that the observed suppression of GFP fluorescence resulted from the degradation of the target-specific GFP mRNA, we measured the level of intracellular mRNA expression using reverse transcriptase-polymerase chain reaction (RT-PCR) for the HeLa-GFP cells grown on the (siRNA/PLL)_n_ multilayers (Fig. 5b). The band intensity of the GFP/β-actin mRNA was 106.54 ± 3.25% for the cells loaded on the PDA-coated substrates, while the (GFP siRNA/PLL)_n_ multilayers showed 90.12 ± 2.31% (n = 1), 64.54 ± 5.56% (n = 3) and 32.56 ± 3.21% (n = 6) for the cells loaded on the (GFP siRNA/PLL)_n_ multilayers. However, the (scrambled siRNA/PLL)_n_ multilayers exhibited no significant degradation of the target mRNA without increasing the layer number under the same experimental conditions. It should be noted that the target-specific gene silencing efficiencies were consistent with the corresponding reduction in the GFP fluorescence of the cells on the (siRNA/PLL)_n_ multilayer (Fig. 4). Interestingly, (GFP siRNA/PLL)_n_ multilayers coated with PLL at the last layer showed enhanced gene silencing as compared to siRNA-coated multilayers, which might be related to the siRNA protection by PLL outer coating from serum degradation (Fig. S6). These results indicate that the gene silencing of the GFP expression in the cells loaded on the siRNA/PLL multilayer was directly induced by the degradation of the target GFP mRNA via the RNAi processing.

## Conclusions

We developed the polyelectrolyte multilayer of siRNA and PLL through LBL self-assembly on the PDA-coated substrate for effective cell adhesion and gene silencing. The surface of substrates was readily coated by PDA through the oxidative self-polymerization of dopamines. The resulting siRNA/PLL multilayer on the PDA-coated substrate exhibited efficient target-specific gene silencing through RNAi activity. Also, the gene silencing efficiency was increased with increasing the number of the siRNA layer in the multilayer. This approach should possess considerable potential for surface-mediated genes delivery.

## Experimental Section

### Materials

All oligonucleotides were purchased from Bioneer Corp. (Daejeon, Republic of Korea). PLL (0.01% w/v, 150–300 kDa) and dopamine hydrochloride were obtained from Sigma-Aldrich (St. Louis, MO, USA). Round cover glasses with a diameter of 18 mm were purchased from Marienfeld-superior (Germany).

### Coating of Polydopamine on the Glass Surface

A glass was thoroughly washed several times with deionized water to eliminate any contaminations. The cleaned glass was immersed in the dopamine solution (2 mg/mL dopamine hydrochloride in 10 mM Tris buffer at pH 8.5). After 48 h incubation, the resultant product, PDA-coated glass, was rinsed 3 times with deionized water and heated at 120 °C for 1 h.

### Construction of siRNA/PLL Multilayers

A siRNA stock solution was prepared at a concentration of 5 μg/mL in 10 mM Tris buffer (pH 7.5). One milliliter of the siRNA solution was dropped on the PDA-coated substrate and incubated for 30 min at room temperature. After washed with deionized water, One milliliter of the PLL solution was dropped on the siRNA-adsorbed PDA-coated substrates for 30 min at room temperature. The sequential deposition of siRNA and PLL was repeated.

### Determination of Loading Efficiency

After the siRNA adsorption process, unattached siRNA was obtained by ethanol precipitation^43^. Briefly, 3 M sodium acetate (pH 5.2) and then 100% ethanol were added to the siRNA solution containing unattached siRNA, followed by incubation at −80 °C for 30 min. The pellets were carefully dissolved in diethyl pyrocarbonate-treated deionized water. The loading efficiency of siRNA molecules was determined at 260 nm using a Nanodrop® ND-1000 Spectrophotometer (Wilmington, DE, USA).

### Gene Silencing

The resultant (siRNA/PLL)_n_ multilayer on the PDA-coated glass was placed carefully at the bottom of the wells in a 12-well plate. HeLa cells expressing GFP (HeLa-GFP cells) was seeded on the surface of siRNA/PLL multilayers at a density of 1.5 × 10^5^ cells per well in a serum-deficient medium. After 6 h incubation, the transfected cells were washed with phosphate buffered saline and further incubated in fresh culture medium for 48 h. The total RNA was extracted from the cell lysates using TRI reagent (Ambion, Inc., USA) and directly transcribed to cDNA using an Ominscript RT-PCR kit (Qiagen, USA) according to the manufacturer’s instructions. The resulting cDNA was amplified using Tag polymerase and its specific primer sets following previous studies^18^. To visualize the suppression of GFP fluorescence, the HeLa-GFP cells were analyzed using confocal laser scanning microscopy (LSM 510, Carl Zeiss). Briefly, the cells grown on the multilayers were washed with PBS and fixed with 3.7 wt% formaldehyde for 10 min at room temperature. The nuclei of the cells were stained using 4′,6-diamidino-2-phenylindole (DAPI, 1.5 mg/mL^−1^) for 5 min.

### Cell Cytotoxicity

HeLa-GFP cells were seeded on the surface of siRNA/PLL multilayers at a density of 1.5 × 10^5^ cells per well in a 12-well plate. After 6 h incubation, the transfected cells were washed with PBS and further incubated in a fresh culture medium. After 48 h incubation, the number of viable cells was analyzed using an EZ-Cytox Enhanced Cell Viability Assay Kit (Daeillab service co. Ltd., Seoul, Republic of Korea).

### Characterization of siRNA/PLL Multilayers

The surface of various (siRNA/PLL)_n_ multilayers was observed using scanning electron microscopy (SEM, S-4800, Hitachi Ltd., Japan). The specimen was sputter-coated with platinum for 120 seconds. The loading of siRNA was confirmed by preparing a polyelectrolyte multilayer with TAMRA-labeled siRNA, followed by visualization using confocal laser scanning microscopy (LSM 510, Carl Zeiss).

### Statistical Analyses

All data are presented as the mean standard deviation of n independent measurements. Statistical significance was determined for p < 0.05 using the Student’s test.

## Electronic supplementary material

supplementary information
