# Supplementary material for: Layer-by-layer siRNA/poly(L-lysine) Multilayers on Polydopamine-coated Surface for Efficient Cell Adhesion and Gene Silencing
Source: Sci Rep. 2018 May 17;8:7738. doi: 10.1038/s41598-018-25655-7 (PMC5958135; doi:10.1038/s41598-018-25655-7)
Supplement: Supplementary file 1 — supplementary information [file 41598_2018_25655_MOESM1_ESM.docx]

**Supplementary Information**

**Layer-by-layer siRNA/poly(L-lysine) Multilayers on Polydopamine-coated Surface for Efficient Cell Adhesion and Gene Silencing**

**Cheol Am Hong^1^, Ho Yeon Son^1^, and Yoon Sung Nam^1,2,*^**

^1^Department of Materials Science and Engineering and ^2^KAIST Institute for the NanoCentury, Korea Advanced Institute of Science and Technology, Daejeon 34141, Republic of Korea

* To whom all correspondence should be addressed.

E-mail: yoonsung@kaist.ac.kr; phone: +82-42-350-3311; and fax: +82-42-350-3310


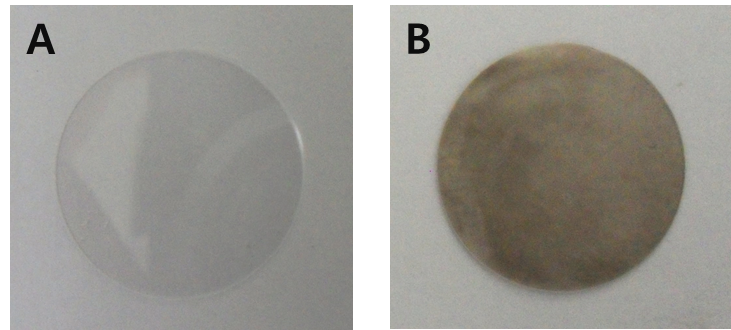


**Figure S1**. Photograph of a bare glass surface before (A) and after (B) PDA coatings via the oxidative polymerization of dopamines.

**Figure S2.** SEM image of the (siRNA/PLL)_6_ multilayer on the PDA-coated glass substrates and pristine glass surface. The dotted black line marks the separation between PDA-coated glass substrates (left side) and glass surface (right side). Scar bar = 200 μm.

**Figure S3.** Cross-sectional SEM image (a) and thickness (b) of the (siRNA/PLL)_n_ multilayers on the PDA-coated glass substrates. (a) Arrow indicates (siRNA/PLL)_6_ multilayers .Scale bar = 100 μm.

**Figure S4.** 1 % agarose gel images (a) and quantitated analysis (b) of the released siRNA from (siRNA/PLL)_6_ multilayers on the PDA-coated glass substrates after incubation of 0, 3, and 5 days.

**Figure S5**. Confocal microscopy images (a) and the density (b) of the HeLa-GFP cells attached on the pristine PDA-coated glass surface and (siRNA/PLL)_n_ multilayers on the PDA-coated substrates after 3h incubation. PDA-coated glass surface (A), (siRNA/PLL)_2_ (B), (siRNA/PLL)_4_ (C), and (siRNA/PLL)_8_ (D) on the PDA-coated glass substrates. Scar bar = 50 μm. The density of adherent cells was calculated by counting the DAPI-stained cells using Images J.

******

**Figure S6.** Gene silencing effect of the HeLa-GFP cells grown on the (siRNA/PLL)_n_ multilayers (n = 1, 3, and 6) coated with PLL and siRNA at the last layer. ***P* <0.05
